# Quantification of Hydrogen Peroxide in PVP and PVPVA Using ^1^H qNMR Spectroscopy

**DOI:** 10.3390/polym17060739

**Published:** 2025-03-11

**Authors:** Isha Saraf, Varun Kushwah, Bernd Werner, Klaus Zangger, Amrit Paudel

**Affiliations:** 1 Research Center Pharmaceutical Engineering GmbH, Inffeldgasse 13, 8010 Graz, Austria; varun.kushwah@cdri.res.in; 2 Institute of Chemistry, University of Graz, Heinrichstr. 28, 8010 Graz, Austria; bernd.werner@uni-graz.at (B.W.); klaus.zangger@uni-graz.at (K.Z.); 3 Institute of Process and Particle Engineering, Graz University of Technology, Inffeldgasse 13, 8010 Graz, Austria

**Keywords:** reactive impurities, ^1^H qNMR, peroxides, excipient, polymer

## Abstract

Objective: Peroxides in pharmaceutical products and excipients pose risks by oxidizing drug molecules, leading to potential toxicity and reduced efficacy. Accurate peroxide quantification is essential to ensure product safety and potency. This study explores the use of quantitative proton nuclear magnetic resonance (^1^H qNMR) spectroscopy as a sensitive and specific method for quantifying peroxide levels in pharmaceutical excipients. Methods: ^1^H qNMR spectroscopy was employed to measure peroxide levels down to 0.1 ppm in excipients, focusing on poly(vinylpyrrolidone) (PVP) and polyvinylpyrrolidone/vinyl acetate (PVPVA). Different grades and vendors were analyzed, and the impact of various manufacturing processes on hydrogen peroxide content was examined. Results: Peroxide levels varied among different grades of PVP and PVPVA, as well as between vendors. Furthermore, manufacturing processes influenced the hydrogen peroxide content in selected excipients. These variations highlight the importance of controlling peroxide levels in raw materials and during production. Conclusions: ^1^H qNMR spectroscopy is a valuable tool for accurately quantifying peroxide levels in pharmaceutical excipients. The study emphasizes the need for regular monitoring of peroxide content to ensure the stability, quality, and safety of excipients and drug products. Accurate peroxide measurement can prevent oxidative degradation, preserving both the safety and efficacy of pharmaceutical formulations.

## 1. Introduction

Excipients are used in pharmaceutical products to ameliorate the biopharmaceutical performance of the API [1]. Primarily, pharmaceutical excipients are inert and thus considered as GRAS (generally regarded as safe) substances [2]. However, owing to numerous processing steps involved in their synthesis or drug product processing, they often contain trace levels of reactive impurities, such as hydrogen peroxides [3,4,5].

Hydrogen peroxides are a class of highly reactive and potentially hazardous oxygen species that can cause unwanted reactions and thereby the degradation of active ingredients in pharmaceutical products (API) [6]. Even present in trace levels, hydrogen peroxide can act as a free radical generator and/or oxygen donor and it reacts with API to form degradation products during the manufacturing and/or storage conditions [3,7,8]. Therefore, it is important to monitor and quantify the concentration of peroxides in pharmaceutical products and excipients. Various analytical techniques have been developed for peroxide quantification, including the use of the methods based on titration, colorimetry, and chemiluminescence [9,10,11,12,13]. However, these methods are often time-consuming, require extensive sample preparation, and lack the sensitivity and specificity required for accurate peroxide quantification. Some of these colorimetric methods are also included in the pharmacopoeal monographs of the respective excipients, wherein the typical permitted limit of peroxide is stated in the range of 400 ppm [14,15,16,17]. However, there are documented cases of drug oxidation occurring in PVP containing formulations even at the peroxide content below the pharmacopoeal limit [18]. This strongly justifies the necessity of a more sensitive and specific peroxide quantification method [19]. Furthermore, conventional methods for hydrogen peroxide quantification, such as titration and chromatography, often require complex sample preparation, derivatization, or indirect detection techniques, which may introduce variability and matrix interferences. In contrast, our NMR-based approach offers a direct, non-destructive, and highly specific means of quantifying hydrogen peroxide without the need for chemical modifications or extensive sample manipulation. By leveraging the distinct proton signals of H_2_O_2_ in the NMR spectrum, our method provides quantitative accuracy, structural insights, and compatibility with polymer matrices, making it a powerful alternative to traditional analytical techniques.

In recent years, proton nuclear magnetic resonance spectroscopy (^1^H NMR) has emerged as a promising analytical tool for the quantification of excipients in pharmaceutical products due to its high sensitivity, specificity, and ease of use [20,21,22,23,24]. ^1^H NMR is a non-destructive and non-invasive analytical technique that measures the signal intensity of protons in a sample. The signal intensity is proportional to the concentration of the nuclei of the analyte [24,25,26]. Currently, the ^1^H NMR method is widely used for the excipient analysis and quantification. In a study titled “Quantitative Chemical Profiling of Commercial Glyceride Excipients via ¹H NMR Spectroscopy”, our lab developed a method to quantify the chemical composition of glyceride-based excipients. This approach enabled the identification and quantification of various glycerides, providing insights into the quality and consistency of these excipients used in pharmaceutical formulations.

Additionally, in the research “Interplay of Aging and Lot-to-Lot Variability on the Physical and Chemical Properties of Excipients: A Case Study of Mono- and Diglycerides”, the effect of aging and batch variability on the physical and chemical properties of mono- and diglycerides was investigated. Utilizing ¹H NMR spectroscopy, the study correlated changes in chemical composition with alterations in the solid-state characteristics of these excipients, emphasizing the importance of monitoring such variations to ensure product stability and performance. In this context, the ^1^H NMR method can be developed for the quantitative determination of the peroxide concentration in the sample. ^1^H NMR has several advantages over traditional peroxide quantification methods. It does not require the use of hazardous reagents or extensive sample preparation, which can reduce the risk of sample contamination or loss [27]. Additionally, the technique provides a rapid and reliable measurement of peroxide concentration, which can aid in the quality control and optimization of pharmaceutical products and excipients’ grades, suppliers, and storage [28,29,30,31]. Therefore, ^1^H NMR is a promising analytical tool for peroxide quantification in the pharmaceutical industry.

In this manuscript, we present a comprehensive guide for the quantitative determination of peroxides in pharmaceutical products and excipients using ^1^H NMR spectroscopy. The commonly used pharmaceutical excipient polymers, i.e., poly(vinylpyrrolidone) (PVP) and polyvinylpyrrolidone/vinyl acetate (PVPVA) were selected as model excipients. PVP and PVPVA are the polymers of the monomer N-vinylpyrrolidone and the 6:4 linear random copolymer of N-vinylpyrrolidone and vinyl acetate, respectively. PVP and PVPVA 64 are available from different vendors, like Ashland and BASF. PVP grades of varying chain lengths and PVPVA of different VP to VA ratios can be found. Furthermore, an ultra-grade of PVPVA is also available from Ashland, which is reported to contain significantly lower levels of hydrogen peroxide compared to the normal grade available [19,32,33,34,35].

Hydrogen peroxide is used in the synthesis of both the PVP and PVPVA polymers and the levels of hydrogen peroxide regulates the level of polymerization [36]. After the synthesis, the excipients were purified to remove any unreacted hydrogen peroxide impurity. In spite of extensive purification steps, PVP and PVPVA contain trace level of hydrogen peroxide in the final product, responsible for poor pharmaceutical product quality [32,37].

In the present work, the peroxide content was quantified in different grades, as were lots of PVP and PVPVA from different vendors. Furthermore, in order to evaluate the effect of typical drug product process conditions, the PVPVA polymer was hot melt extruded and milled using a hot melt extruder (HME) and ball mill, respectively. Thereafter, the samples were analyzed for their hydrogen peroxide levels. Different grades of PVPVA samples from BASF and Ashland were extruded and milled and evaluated for the change in hydrogen peroxide levels. Furthermore, a design of experiments (DoE) study was conducted to evaluate the effect of particle size of PVPVA, temperature, and humidity on the hydrogen peroxide levels of the extruded and milled PVPVA ultra-grade sample from Ashland (PVPVA-U-A). Thus, the present manuscript deals with novel method development for the quantitative analysis and evaluates the effect of different pharmaceutical processes on the levels of hydrogen peroxide. Our work provides a valuable resource for researchers and analysts in the pharmaceutical industry for rapid and precise measurement of peroxide levels.

## 2. Materials

Different lots and grades of polyvinylpyrrolidone (PVP) were procured from Sigma (Vienna, Austria), Ashland (Düsseldorf, Germany), and BASF (Burgbernheim, Germany) whereas different grades and lots of copovidone (PVP-VA) were purchased from Ashland (Düsseldorf, Germany) and BASF (Burgbernheim, Germany).

Hydrogen peroxide was procured from Sigma (Vienna, Austria). All other chemicals used were of analytical grade. The nomenclature of PVP grades and lots used hereafter is listed in Table 1.

## 3. Methods

### 3.1. Peroxide Quantification Using ^1^H qNMR

The peroxide quantity was quantified using ^1^H-NMR. For this 25.0 mg of excipient, 1 mL of DMSO containing 100 µg of tCNB was used as a reference standard. Finally, the solution was transferred into NMR tubes (Eurisotop, Essonne, France) and the ^1^H-NMR spectra were acquired. Furthermore, the amount of peroxides in the samples was quantified using the calibration curve method. Briefly, 7-point calibration with different concentrations of hydrogen peroxide in the range of 0.1 to 10 ppm containing the tCNB reference standard were prepared. Thereafter, the solution was transferred into NMR tubes (Eurisotop, France), and the ^1^H-NMR spectra were acquired.

#### 3.1.1. Standards and Mixtures

**Figure 1:**
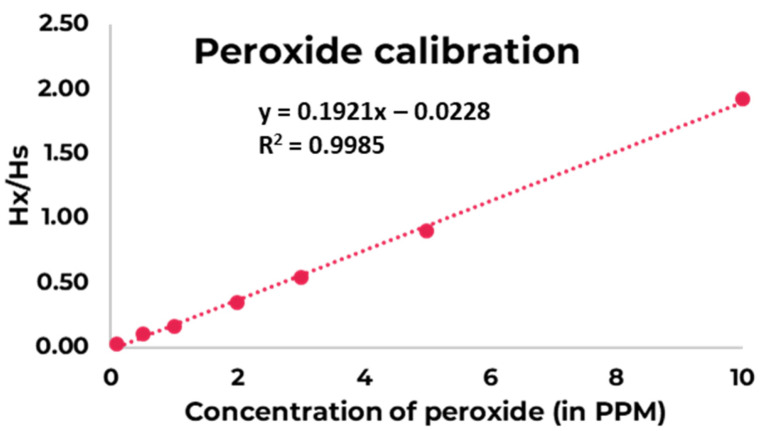
Calibration curve of the hydrogen peroxide.

For the method development, 1,2,4,5-tetrachloro-3-nitrobenzene (tCNB), (Sigma Aldrich, Vienna, Austria) was used as a reference standard for ^1^H-qNMR analysis. For the calibration, different concentrations of PVP and PVPVA were analyzed using ^1^H-qNMR (Figure 1). The quantity of the peroxide was determined in the sample. Deuterated DMSO (Eurisotop, France) was used as an NMR solvent. All the other chemicals and solvents used were of analytical reagent grade.

#### 3.1.2. Equipment

The ^1^H-NMR spectra were recorded on a Bruker Avance III™ HD 500 MHz NMR spectrometer using a 5 mm TCI cryo probe at 298 K. The spectra were processed using MestreNova software (version number 14.3.3).

#### 3.1.3. Method Development for Quantitative ^1^H-NMR (^1^H-qNMR)

**Figure 2:**
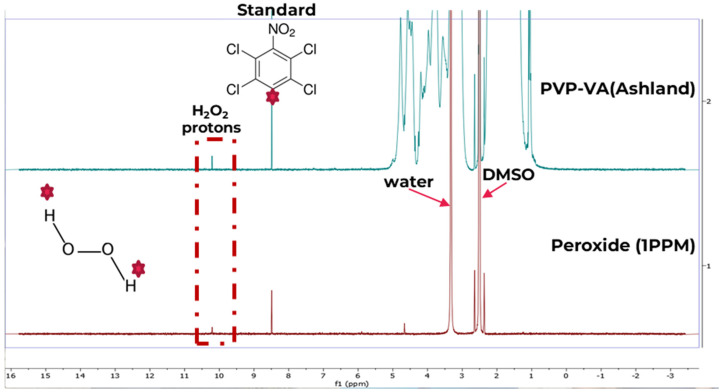
^1^H-NMR spectra of peroxide in hydrogen peroxide and PVPVA samples. (pink hexagram represents the selected protons for the analysis).

Initial experiments were conducted to identify the suitable instrumental parameters (range of relaxation delays and acquisition times) required to obtain accurate quantitative results. After optimization, the acquisition parameters selected were: relaxation delay set to 3 s, scans 64, spectral width 10,000.0 Hz, acquisition time 3.2768 s, and pulse width 45°. The raw data were multiplied with an exponential window function using a line broadening of 3 Hz. The spectra were baseline corrected and the chemical shift of the spectra was calibrated using chemical shift of the tCNB reference standard (at 8.47 ppm, one aromatic proton). The signal of the -OH proton (at ~10.3 ppm) was selected for the quantification of peroxide content. Each spectrum was recorded in triplicate and the data provided were average values with the standard deviations. The quantity of the peroxide in the samples were calculated using the calibration curve plotted between the ratio of Hx/Hs and the concentration of the peroxide. Here, Hx and Hs were the integral value of selected signals of analyte and that of selected signals of the standard, respectively (Figure 2, and Table 2). Furthermore, additional comparative analyses were performed using a Pierce™ Quantitative Peroxide Assay Kit (Thermo Scientific Pierce, Rockford, IL, USA) and compared with the NMR results for the quantification of H_2_O_2_ in 30% H_2_O_2_ solution, in order to verify the developed method.

### 3.2. Method Verification Using Commercial Samples

#### 3.2.1. PVP and PVPVA Raw Material

##### Lot-to-Lot Variation

The quantity of the hydrogen peroxide present in the excipients were investigated using the developed ^1^H NMR method. Different lots of PVPs and PVPVA (Table 1) from various vendors were investigated for the lot-to-lot variability. Briefly, the raw materials (PVP and PVPVA), without undergoing any further treatment, were dissolved in deuterated DMSO along with the 1,2,4,5-tetrachloro-3-nitrobenzene (tCNB) standard and analyses using a 500 MHz NMR spectrometer. Three PVP grades from Sigma, i.e., K30, K60 and K90, and two commercially available PVP grades from Ashland and BASF, i.e., K30 and K90, were studied. Furthermore, the hydrogen peroxide was also evaluated in two PVPVA grades from Ashland and one PVPVA grade from BASF.

#### 3.2.2. PVPVA Sample Treatment

##### Hot Melt Extrusion (HME)

**Figure 3:**
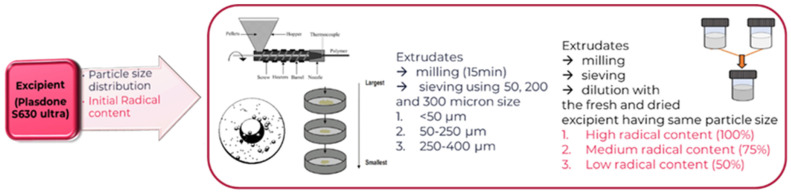
Input factors for the DoE studies and study design.

The combined effect of temperature and shear stress was evaluated using HME. The extrudates of the PVPVA (PVPVA-U-A-1, PVPVA-A-1 and PVPVA-B-1) were prepared using a Three Tec ZE9 HMI table top 9 mm twin-screw mini extruder (Three-Tec, Seon, Switzerland) with a die size of 1.5 mm. A conveying belt (with 10-level speed range: 1.72–6.06 m/min) was used to cool the strands during conveying at ambient temperature and humidity. The temperature of the three barrels was set to 80° (first zone close to feeder), 120°, and 120 °C starting with the first heating zone, whereas, the screw speed was kept constant to 100 rpm. The samples were collected and analyzed for the hydrogen peroxide content using the developed method (Figure 3).

##### Ball Mill to Generate Powder from Excipient Extrudate

The extrudates of the PVPVA (PVPVA-U-A-2) obtained were then milled using a ball mill. The milling experiments of the PVPVA samples were performed at an ambient temperature using a Retsch Mill (Retsch, GmbH, Haan, Germany). Briefly, 1 g of different PVPVA extrudates were milled in a 50 mL container having a 2 cm stainless steel ball. The ball mill was operated at the frequency of 25 Hz. Furthermore, in order to generate the different particle sizes of the samples, the extruded PVPVA samples were milled for 15 min and sieved using a 50, 250 and, 400 µm mesh size to obtain particle size in the range of <50, 50 to 250 and 250 to 400 µm. Thereafter, all the samples were analyzed by the developed ^1^H NMR method and also stored under ambient conditions for one month.

##### Storage Stability

The effect of storage conditions, time, and initial free radical concentration on the hydrogen peroxide levels in the PVPVA-U-A-2 samples (Table 1) was evaluated. Briefly, the samples (milled samples, from Section 3.2.2) were kept in different conditions of temperature and humidity of 25, 40, and 70 °C and 20, 45, and 75%RH, respectively, for up to 90 days (Figure 3, Table 3 and Table 4).

#### 3.2.3. Design of Experiments (DoE): Impact of Process Conditions on the Hydrogen Peroxide Content in Micronized Extruded PVPVA-U-A-2

D-optimal designs are provided by a computer algorithm and can be used to fit different types of model (first and second orders, quadratic, cubic) or for objective-like screening or generating a response surface. Furthermore, D-optimal designs are very handy when the classical designs are not implemented or when limiting the number of experimental trials to provide a constrained design space. The experimental design and statistical analysis were performed using the MODDE^®^-Design of Experiments Software (MODDE 10, Umetrics, Umea, Sweden). A four-factors-three-levels experimental design was used to evaluate the effect of independent variables like temperature, humidity, particle size of PVPVA, and the initial free radical content of the excipient (PVPVA). The samples with different particle sizes were prepared as mentioned above in Section 3.2.2. Furthermore, to obtain different levels of initial free radicals content, the milled samples (with the highest levels of free radicals) were diluted with an untreated sample of the same particle size. The samples were also placed at different time points (3, 14, and 28 d) for the evaluation of kinetic behavior (Figure 3). Thus, a total of 42 samples were prepared for six time points, whereas, 9 samples of d 0 were also evaluated. The details of the excipients are mentioned in Table 4.

## 4. Results and Discussion

### 4.1. Method Verification Using Commercial Samples

#### 4.1.1. PVP and PVPVA Raw Material

##### Lot-to-Lot Variation

For the analysis of hydrogen peroxide in pharmaceutical polymers, we selected DMSO-d_6_ as the aprotic solvent due to its non-overlapping peak with the signals of interest, unlike other solvents, such as CDCl_3_ and DMF, which can interfere with polymer signals. This makes DMSO-d_6_ particularly suitable for polymeric analysis. Additionally, the use of tCNB as an internal standard in our study further enhances accuracy, as its distinct, non-overlapping peak allows for reliable H_2_O_2_ quantification in polymer matrices.

**Figure 4:**
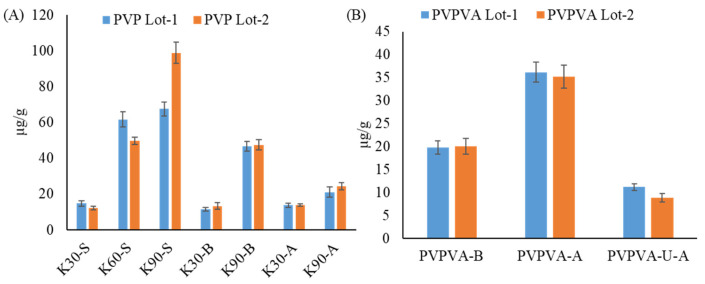
Hydrogen peroxide levels in different lots and grades of (**A**) PVPs and (**B**) PVPVA from different vendors.

The reactive impurity (hydrogen peroxide) levels were quantified for fresh polymers (PVP and PVPVA) using the ^1^H qNMR method. In all the samples, no change in the pH was observed on dissolving the polymer (PVP-VA) in water, which could have resulted in a change in peroxide level affecting the developed ^1^H qNMR method. For the PVPVA samples, the amounts of hydrogen peroxide were found to be different in case of different vendors and also different grades. The hydrogen peroxides were found to be higher in the case of PVPVA from Ashland as compared to BASF. However, the ultra-grade from Ashland showed markedly lower levels of hydrogen peroxides as compared to the normal grade of Ashland and BASF (Figure 4) [38,39,40].

In the case of the PVP polymer, the level of peroxide in the samples varied with different vendors and grades (Figure 4). However, negligible lot-to-lot variation was observed, except for the Sigma grade. Interestingly, the peroxide level was found to be higher in the case of the long chain polymer (PVP 90) as compared to the short chain polymer (PVP K30). Furthermore, in contrast to the PVPVA, higher levels of peroxides were found in the case of PVP K90 polymers from BASF as compared to the PVP K90 polymer from Ashland, whereas, no marked difference was observed in the peroxide levels in the case of the PVP K30 polymer from both Ashland and BASF. The hydrogen peroxide levels in Sigma K60 and K90 samples were found to be higher as compared to both Ashland and BASF. As a result of different numbers of reactive impurities (hydrogen peroxide), the ultra-grade of the PVPVA could be useful in developing formulations with API susceptible to oxidative degradation [39,40].

#### 4.1.2. PVP-VA Sample Treatment

In order to evaluate the effect of shear stress and temperature, different grades of PVPVA from different vendors were extruded and micronized using a ball mill. Thereafter, the samples were kept at room temperature for one month. The fresh micronized and stored samples were then evaluated for the hydrogen peroxide levels using the developed method. Two grades (Lot-1), i.e., normal and ultra-grades of PVPVA, were investigated from Ashland, whereas, a normal grade (Lot-1) of PVPVA from BASF was used.

**Figure 5:**
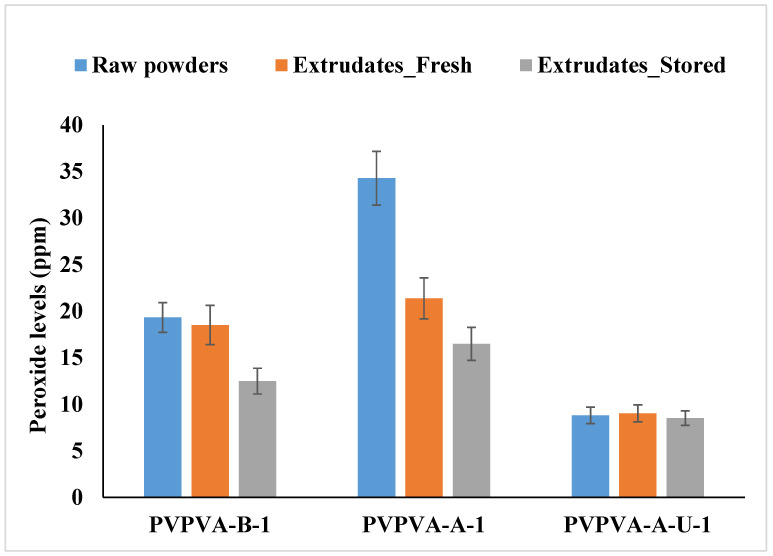
Peroxide levels in fresh powder, fresh extrudates, and extrudates stored at RT for one month.

The extruded samples showed a decrease in the hydrogen peroxide level in the case of PVPVA-A-1, whereas comparable peroxides were found in the case of PVPVA-B-1 and PVPVA-A-U-1 as compared to the raw counterparts. Interestingly, the levels of peroxide decreased in the cases of both PVPVA-B-1 and PVPVA-A-1 after storing the samples at RT for one month. A marginal decrease was observed in the case of PVPVA-A-U-1 (Figure 5).

Hydrogen peroxide is retained in the powder by forming a complex with the polymer. This complexation prevents the hydrogen peroxide from being in a free phase, which would otherwise lead to its rapid evaporation [32]. By binding with the polymer, the hydrogen peroxide remains stable and can be effectively utilized in its intended application without significant loss due to evaporation [41]. This method ensures a controlled release and enhances the efficacy and shelf-life of products containing hydrogen peroxide. In the case of extrusion samples, in some case, due to changes in the molecular dispersity, peroxide may not be bound to powder, resulting in the escape of hydrogen peroxide from the sample [32]. Moreover, upon storage, peroxide might be consumed via polymer oxidation in extrudate samples due to the activation of chains during the extrusion process [42,43,44].

#### 4.1.3. Design of Experiments (DoE): Impact of Process Conditions on the Hydrogen Peroxide Content in Micronized Extruded PVPVA-U-A-2

The significant factors influencing the hydrogen peroxide content were determined using the nominal levels model coefficient plot. The particle size distribution of the excipients (positive coefficient) had the highest impact, indicated by the magnitude of coefficients on the response, followed by the humidity (negative coefficient), temperature (positive coefficient), and the initial free radical content (negative coefficient). Overall, particle size distribution of excipient and humidity were found to be the critical factors affecting peroxide level in the samples.

The entire data analysis resulted in the following regression equations, which suggests an empirical relationship between the response values and the independent variables.

Peroxide level (µg/g) = 2.84125 + 0.852375 × Particle size distribution of excipients [µm] − 0.540954 × Humidity [%] + 0.384624 × Temperature [°C] − 0.281643 × Initial free radical content(1)

The peroxide levels were found to be lower in the case of samples with higher free radicals, which could be due to the interaction of initial free radicals creating peroxide radicals in the case of fresh samples (d = 0). Furthermore, the peroxide level was found to be higher in the case of samples stored in conditions with a higher temperature and lower humidity, due to the amplification of the peroxide radicals (at higher temperature) and higher stability (at lower humidity, i.e., not decomposed into hydrogen and water) (Table 4).

**Figure 6:**
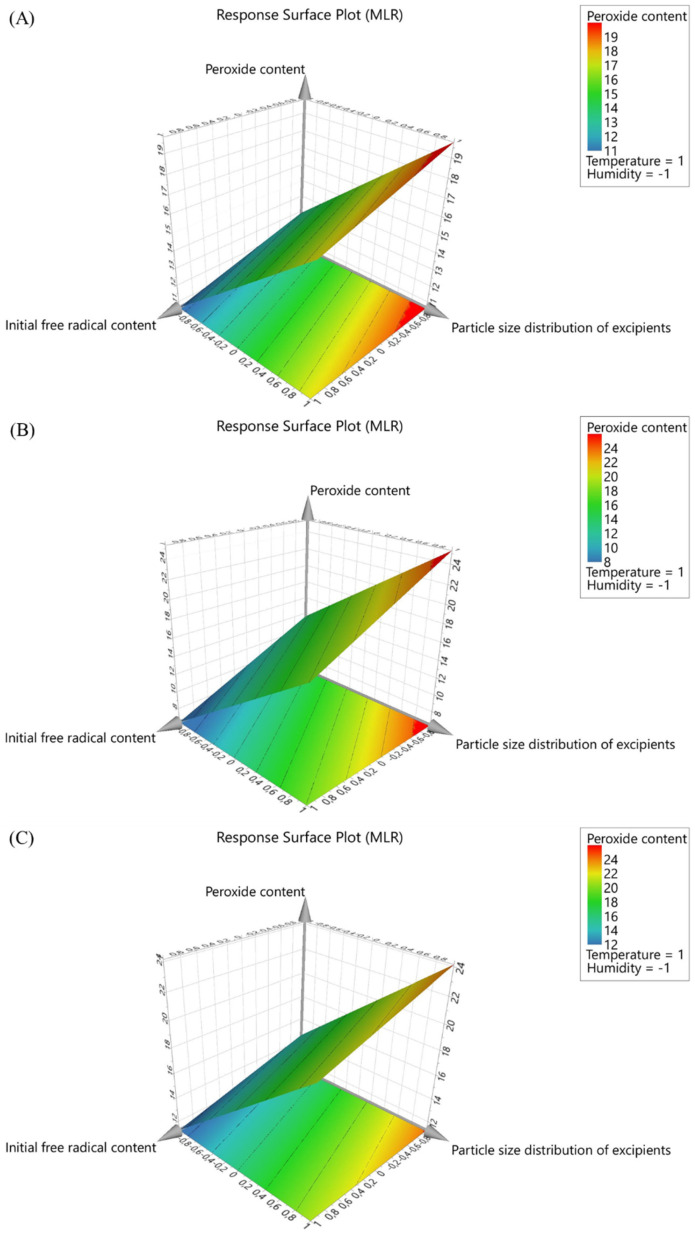
Response surface plot depicting interaction of input parameters on the peroxide level at day (**A**) 3, (**B**) 14, and (**C**) 28.

Interestingly, the levels of peroxide were found to increase with a higher particle size of the excipient, which could be due to the lower propensity of the large particles to absorb moisture due to a lower surface area [45,46]. Lower levels of moisture could lead to lower levels of hydrogen peroxide decomposition into water and hydrogen resulting in higher hydrogen peroxide stability [47,48]. The level of hydrogen peroxide was found to be in the following order, 250 to 400 > 250 to 50> less than 50 µm of excipient particle size (Figure 6, Table 4). In the case of the storage samples (Figure 6), the effect of initial free radical content was found to be marginally lower, which could be due to the short half-life of free radicals in storage conditions.

## 5. Industrial Applicability

The ^1^H NMR method for peroxide quantification has several advantages over traditional methods that are currently used in the pharmaceutical industry. One of the primary advantages is the speed and simplicity of the method, which allows for high-throughput sample analysis and streamlined quality control processes. Additionally, the non-destructive nature of ^1^H NMR analysis allows for repeated measurements of the same sample, enabling better statistical analysis and minimizing the need for additional samples. The method is also more cost-effective than traditional methods, as it does not require the use of hazardous reagents or specialized equipment.

The industrial application of the ^1^H NMR method for peroxide quantification can have a significant impact on the quality and safety of pharmaceutical products [34,49,50,51,52]. By accurately measuring the peroxide levels in a range of products (raw materials, intermediate, and final pharmaceutical products), manufacturers can ensure that their products are safe for human use and meet regulatory standards (lower API degradation). The method can also be used to assess the stability of pharmaceutical formulations over time, enabling manufacturers to optimize their products and improve their shelf-life. Furthermore, the use of the ^1^H NMR method for peroxide quantification can aid in the development of new pharmaceutical products, providing a reliable and efficient means of screening and optimizing formulations.

Overall, the industrial application of ^1^H NMR method for peroxide quantification in pharmaceutical products and excipients has significant potential to improve the safety, efficacy, and efficiency of the drug development process. The method provides a non-destructive, non-invasive, and cost-effective approach to measuring peroxide levels in a variety of samples, enabling high-throughput analysis and streamlined quality control processes. As such, the ^1^H NMR method has become an essential tool for researchers and analysts in the pharmaceutical industry seeking to ensure the safety and efficacy of their products.

## 6. Conclusions

The peroxides were quantified using the ^1^H qNMR method. The developed method was found to be effective in a quantity of a lower amount of (in ppm) peroxides. Interestingly, the reactive impurities present in PVP and PVPVA were found to be different many folds with different grades and from different vendors, which can affect the quality of pharmaceutical products. Furthermore, the peroxide content in the case of PVPVA treated with manufacturing processes commonly used in the pharmaceutical industry were also evaluated. The temperature, humidity, and physicochemical properties such as particle size distribution were found to be the most critical parameters of the concentration of peroxides generated after the process treatment. Thus, this also showcases the importance of lot-to-lot variability and possibly also vendor-to-vendor variability in residual impurity levels of excipients. The present investigation also accounts for the development of excipient guidelines with stringent specification levels of impurities, especially for the development of next-generation or complex formulations.

In conclusion, peroxide quantification is a critical step in ensuring the safety and efficacy of pharmaceutical products and excipients. The use of proton nuclear magnetic resonance spectroscopy (^1^H NMR) provides a promising method for the quantitative determination of peroxides due to its high sensitivity, specificity, and ease of use. Compared to traditional peroxide quantification methods, ^1^H NMR offers several advantages, including non-destructive and non-invasive sample analysis, reduced sample preparation time, and the absence of hazardous reagents. This manuscript provides a comprehensive guide for the reliable and accurate quantification of peroxides in pharmaceutical products and excipients using ^1^H NMR, which can aid in the quality control and optimization of pharmaceutical formulations. The availability of this method and its implementation by the pharmaceutical industry could contribute to the development of safer and more effective pharmaceutical products.

## Figures and Tables

**Table 1:** Different lots of PVPs and PVPVA used.

	Polymer Grades	Lot 1	Lot 2
PVP	K-30_Sigma	K30-S-1	K30-S-2
	K-60_Sigma	K60-S-1	K60-S-2
	K-90_Sigma	K90-S-1	K90-S-2
	K-30_BASF	K30-B-1	K30-B-2
	K-90_BASF	K90-B-1	K90-B-2
	K-30_Ashland	K30-A-1	K30-A-2
	K-90_Ashland	K90-A-1	K90-A-2
PVPVA	BASF	PVPVA-B-1	PVPVA-B-2
	Ashland	PVPVA-A-1	PVPVA-A-2
	Ultra_Ashland	PVPVA-U-A-1	PVPVA-U-A-2

**Table 2:** Amount of standard hydrogen peroxide used for the preparation of peroxide calibration curve.

Sample Nr.	Sample Name	Concentration of Peroxide in 1 mL DMSO (in PPM)	Hx	Hs	Hx/Hs
1	0.00001% *W*/*V* (0.1 PPM)	0.1	2073.4	85,642	0.02
2	0.00005% *W*/*V* (0.5 PPM)	0.5	8469.7	85,165	0.10
3	0.0001% *W*/*V* (1 PPM)	1	13,927	87,264	0.16
4	0.0002% *W*/*V* (2 PPM)	2	29,272	85,345	0.34
5	0.0003% *W*/*V* (3 PPM)	3	44,177	82,343	0.54
6	0.0005% *W*/*V* (5 PPM)	5	75,527	83,576	0.90
7	0.001% *W*/*V* (10 PPM)	10	167,247	86,954	1.92

**Table 3:** Details of DoE levels.

DOE Levels	Excipient (PVPVA-U-A-2)		Stability Conditions	
	PSD	Initial Radical Content	Temperature	Humidity
−1	<50 µm	Low	25 °C	0–20%
0	50–250 µm	Medium	40 °C	45%
1	250–400 µm	High	70 °C	75%

**Table 4:** Details of excipients in DoE.

Control Samples	Excipient (PVPVA-U-A-2)		
	Sample Name	PSD of Excipient	Initial Radical Content in Excipient
Day-0	E1	<50 µm	High
	E2	<50 µm	Medium
	E3	<50 µm	Low
	E4	50–250 µm	High
	E5	50–250 µm	Medium
	E6	50–250 µm	Low
	E7	250–400 µm	High
	E8	250–400 µm	Medium
	E9	250–400 µm	Low
Instability conditions at different points (3, 14, 28, d)	E3_(25C,20RH)	<50 µm	Low
	E9_(70C,20RH)	250–400 µm	Low
	E1_(70C,20RH)	<50 µm	High
	E1_(25C,75RH)	<50 µm	High
	E7_(25C,75RH)	250–400 µm	High
	E3_(70C,75RH)	<50 µm	Low
	E5_(40C,45RH)	50–250 µm	Medium

## Data Availability

The original contributions presented in this study are included in the article. Further inquiries can be directed to the corresponding authors.
